# Inaccurate secondary structure predictions often indicate protein fold switching

**DOI:** 10.1002/pro.3664

**Published:** 2019-06-17

**Authors:** Soumya Mishra, Loren L. Looger, Lauren L. Porter

**Affiliations:** ^1^ Howard Hughes Medical Institute, Janelia Research Campus Ashburn Virginia 20147

**Keywords:** protein fold switching, metamorphic proteins, multifunctionality, secondary structure predictions

## Abstract

Although most proteins conform to the classical one‐structure/one‐function paradigm, an increasing number of proteins with dual structures and functions have been discovered. In response to cellular stimuli, such proteins undergo structural changes sufficiently dramatic to remodel even their secondary structures and domain organization. This “fold‐switching” capability fosters protein multi‐functionality, enabling cells to establish tight control over various biochemical processes. Accurate predictions of fold‐switching proteins could both suggest underlying mechanisms for uncharacterized biological processes and reveal potential drug targets. Recently, we developed a prediction method for fold‐switching proteins using structure‐based thermodynamic calculations and discrepancies between predicted and experimentally determined protein secondary structure (Porter and Looger, Proc Natl Acad Sci U S A 2018; 115:5968–5973). Here we seek to leverage the negative information found in these secondary structure prediction discrepancies. To do this, we quantified secondary structure prediction accuracies of 192 known fold‐switching regions (FSRs) within solved protein structures found in the Protein Data Bank (PDB). We find that the secondary structure prediction accuracies for these FSRs vary widely. Inaccurate secondary structure predictions are strongly associated with fold‐switching proteins compared to equally long segments of non‐fold‐switching proteins selected at random. These inaccurate predictions are enriched in helix‐to‐strand and strand‐to‐coil discrepancies. Finally, we find that most proteins with inaccurate secondary structure predictions are underrepresented in the PDB compared with their alternatively folded cognates, suggesting that unequal representation of fold‐switching conformers within the PDB could be an important cause of inaccurate secondary structure predictions. These results demonstrate that inconsistent secondary structure predictions can serve as a useful preliminary marker of fold switching.

## Introduction

Most structurally characterized proteins perform one well‐defined function supported by one scaffold of secondary structure.^1^ (Microsecond‐to‐millisecond dynamics of protein tertiary structure have been characterized,^2^, ^3^ but secondary structure remodeling is not typically observed.^4^) Recent data show, however, that some proteins substantially remodel their secondary structures and domain organization in response to cellular stimuli, enabling radical functional changes and tight cellular control.^5^ This phenomenon, called fold switching,^6^ can involve structural and functional transformations as drastic as an α‐helical transcription factor morphing into a β‐barrel translation factor.^7^ The structural changes in some fold‐switching proteins are large enough to foster a transition between soluble globular and integral‐membrane forms. For example, chloride intracellular channel protein 1 is a human protein that functions as both a cytosolic glutathione reductase^8^ and a membrane‐inserted chloride channel.^9^ While entire protein domains can switch folds,^7^ current experimental evidence suggests that it is more common for subdomains of larger proteins to switch folds while the remainder maintains its intact original structure. We call the structurally changing subdomains “fold‐switching regions” (FSRs) and the structurally intact remainders “non‐fold‐switching regions” (NFSRs).

Predicting the fold‐switching ability of a given protein region can suggest a mechanism for its function(s) *in situ*, especially when combined with other forms of evidence that the protein is multifunctional, has more than one cellular localization, or is regulated by a specific environmental trigger. Since other types of proteins—both globular and unstructured—can also exhibit these features,^10^, ^11^ predictions that accurately distinguish between fold switchers and non‐fold switchers would be useful. It is not yet possible to correctly make such predictions with confidence, however. Many factors contribute to this shortfall. For example, all available secondary structure prediction methods, the best of which are homology‐based, are currently unable to predict multiple distinct conformations of a sequence. Instead, they return a single prediction for a given amino acid sequence. Furthermore, robust methods for predicting secondary structure from intrinsic protein properties^12^ are not widely available, especially as pertains to segments of secondary structure that fold into both α‐helix and extended β‐strand, also known as “chameleon sequences.”^13^ Additionally, tertiary structure prediction tools that incorporate *de novo* folding elements, such as Rosetta,^14^, ^15^ can return an ensemble of three‐dimensional models. Such ensembles can be viewed either as multiple guesses at the correct structure or as an estimate of the dynamic conformational rearrangements around a core prediction. Even these state‐of‐the‐art algorithms are unable to deal with multiple potentially well‐folded backbone scaffolds, however.^16^

We successfully predicted FSRs by exploiting incorrect predictions of homology‐based secondary structure predictors.^5^ Specifically, we showed that discrepancies between predicted and experimentally determined secondary structures can indicate that a given protein switches folds. These discrepancies arise from an incompatibility between FSRs and secondary structure predictor design. Specifically, FSRs adopt at least two different secondary structures, but homology‐based secondary structure predictors produce only one best‐guess prediction. Thus, these predictors cannot accurately report both conformations accessible to a given FSR. By coupling secondary structure prediction inaccuracies with a structure‐based thermodynamic calculation,^17^ we were able to successfully predict fold switching in 13 proteins, each with one solved structure and experimental evidence for an alternative conformation.^5^ Thus, discrepancies between predicted and experimentally determined secondary structures can contribute (along with thermodynamic calculations and literature evidence) to estimating whether a given amino acid sequence switches folds. In spite of the promise these discrepancies show for indicating the propensity of an amino acid sequence to switch folds, they have not been shown to have statistical power.

Here, we show that the observation from our previous work is a statistically significant result that can productively and confidently suggest whether or not a given protein switches folds. First, we show that secondary structure predictions of FSRs span a distribution of accuracies, and ~70% of them fall short of 80% accuracy, which is the typical estimate of secondary structure prediction accuracies.^18^ Next, we find that incorrect secondary structure predictions are more common within FSRs than within randomly selected NFSRs of similar length. Inaccurately predicted FSR conformers tend to be underrepresented in the Protein Data Bank (PDB), demonstrating that secondary structure predictions are influenced by structural bias within the PDB. Furthermore, we find that low secondary structure prediction accuracies (<60%) are much more common among fold switchers than non‐fold‐switchers. These results have implications for the improvement of secondary structure predictors as well as identification of fold switching in proteins.

## Results

### *Secondary structure prediction accuracies of FSRs span a wide distribution*

**Figure 1:**
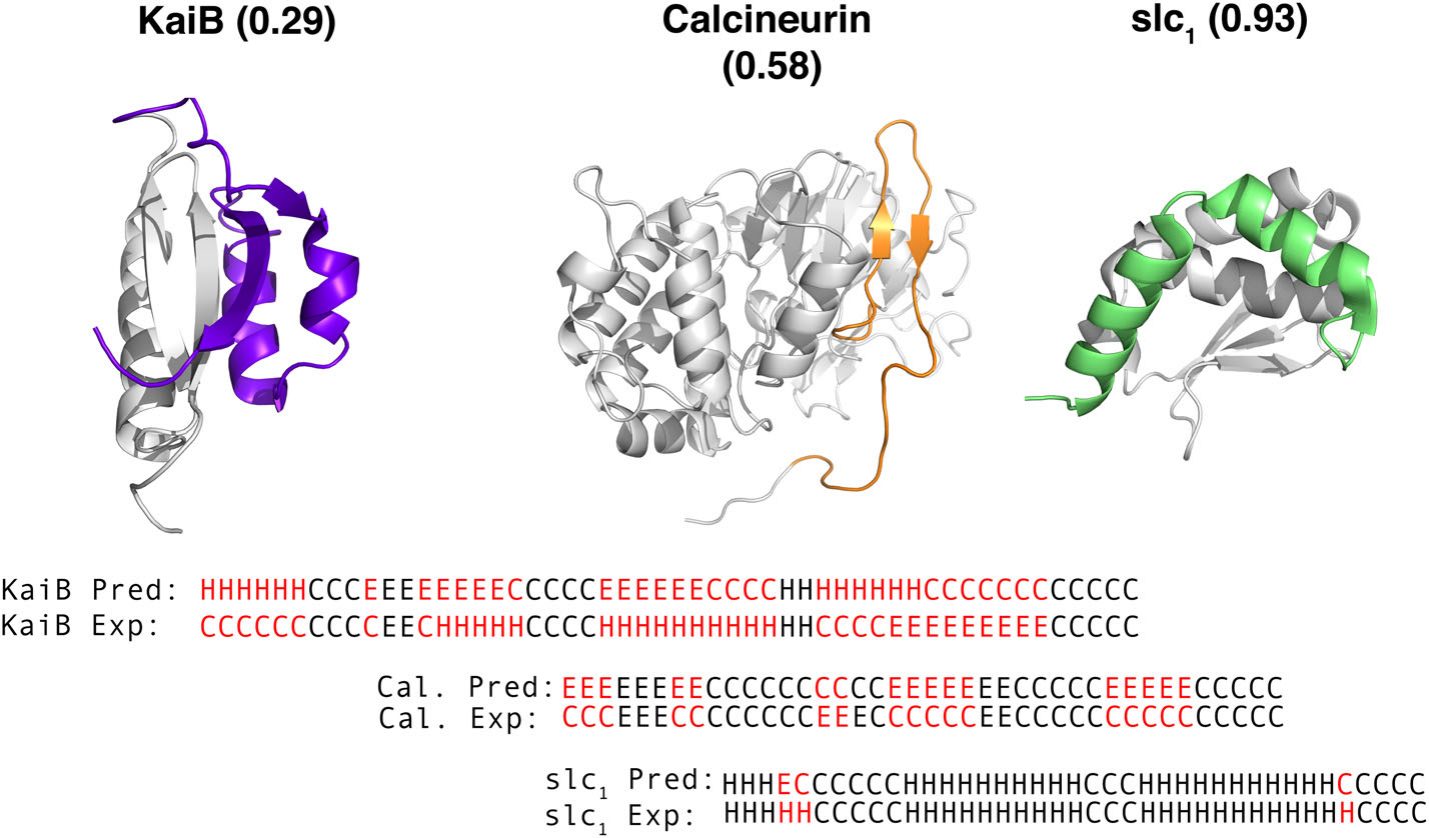
Secondary structure predictions of FSRs span a wide range of accuracies. *Q*_3_ scores range from 0.29 for the inactive tetrameric form of KaiB (purple, pdb ID: 2QKE_A) to 0.58 for the *cis* conformation of calcineurin (orange, pdb ID:5C1V_B), and 0.93 for the monomeric form of archaeal selecase slc_1_ (green, pdb ID: 4QHF_A). Alignments of predicted and experimental secondary structures are shown below protein structures; black letters are consistent; red are inconsistent. Secondary structure predictions were made using JPred4.

**Figure 2:**
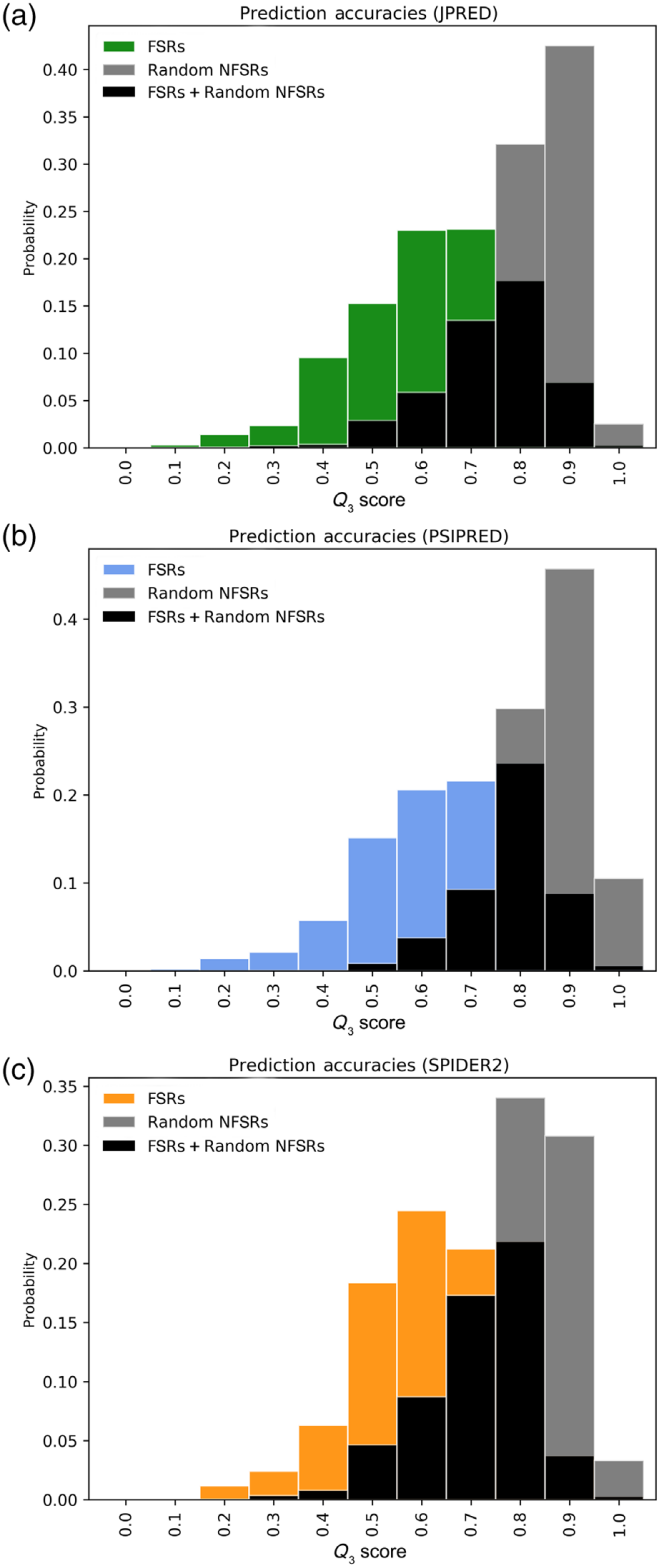
Secondary structure predictions of FSRs are consistently less accurate than those of randomly selected NFSRs. Histograms of fold‐switching fragments are colored (green, JPred; blue, PSIPRED; and orange, SPIDER2), while comparisons of non‐fold‐switching fragments from corresponding predictors are gray; regions of overlap between FSR and random NFSR *Q*_3_ distributions are shown in black.

First, we computed the secondary structure prediction accuracies of FSRs. To do this, we ran three secondary structure prediction software packages (JPred,^19^ PSIPred,^20^ and SPIDER2^21^) on a curated set of 192 structures of fold‐switching proteins^5^ (96 proteins, two structures each). We measured accuracies using the *Q*_3_ metric,^22^ which gives a binary score of 1/0 for agreement/disagreement between the predicted and experimentally determined secondary structure of each amino acid position (Helix, Extended β‐strand, or Coil); this score is summed over sequence and normalized by length. *Q*_3_ scores ranged from inaccurate (0.1) to accurate (1.0) (Figs. 1 and 2). Figure 1 depicts selected examples showing how different *Q*_3_ scores correspond with consistency between prediction and experiment. Secondary structure predictions of tetrameric KaiB, a protein involved in maintaining the rhythm of the cyanobacterial circadian clock,^23^ are inconsistent with experiment (*Q*_3_ = 0.29). As demonstrated from the alignment in Figure 1, one‐third of the discrepancies are H↔E (the most serious error) and the other two‐thirds are C↔H or C↔E. The *cis* conformation of calcineurin's catalytic domain,^24^ a phosphatase that regulates gene expression in response to calcium signals, is an example of moderate agreement between secondary structure predictions and experiment (*Q*_3_ = 0.58). In this case, all disagreements are C↔E. Finally, secondary structure assignments and experiment are in almost perfect agreement for the monomeric form of an archaeal selecase (slc_1_ 25) (*Q*_3_ = 0.93), with only three discrepancies out of 40, two C↔H and one H↔E. Looking at comparisons for all FSRs (Table 1), we found that H↔C discrepancies were most frequent across all predictors: near in value to E↔C discrepancies for JPred and SPIDER2, but different for PSIPRED (H↔C: 50%, E↔C: 38%). In all three cases, H↔E discrepancies were least frequent, ranging from 11% to 15%.

**Table 1:** Frequencies of Secondary Structure Discrepancy in FSRs

	JPred (%)	PSIPRED (%)	SPIDER2 (%)
H↔E	11	12	15
H↔C	45	50	45
E↔C	44	38	40

### *Secondary structure predictions of FSRs are consistently less accurate than those of NFSRs*

Figure 2 shows that secondary structure prediction accuracies for many FSRs are in the low‐to‐moderate range. Approximately 70% of the secondary structure predictions of FSRs from each predictor have *Q*_3_ values <0.8, which is below the typical accuracy of secondary structure predictors.^18^ To determine whether this lower‐than‐expected performance is specific to FSRs or common among all protein regions, we compared the *Q*_3_ distributions of FSRs with randomly selected NFSRs extracted from a curated set of proteins expected to not switch folds.^5^ We found that the secondary structures of these randomly selected NFSRs were predicted much more accurately than for FSRs, with *p*‐values of 1.2 × 10^−264^, 1.6 × 10^−276^, and 3.3 × 10^−176^, respectively (Kolmogorov–Smirnov Test). The average *Q*_3_ for the random NFSRs was 0.83–0.89 (Table 2), in line with typical published accuracies of secondary structure predictors,^18^ while average *Q*_3_s for FSRs were <0.70 for all three predictors. Together, these results indicate that secondary structure prediction accuracies of FSRs are significantly worse than the accuracies of NFSRs, using three state‐of‐the‐art homology‐based secondary structure prediction algorithms. Furthermore, comparisons of these distributions suggest that secondary structure predictions with accuracies <0.60 could be reasonable preliminary indicators of fold‐switching proteins (Fig. 2).

**Table 2:** Mean and Median Secondary Structure Prediction Accuracies

	JPred mean/median	PSIPRED mean/median	SPIDER2 mean/median
FSRs	0.67/0.69	0.68/0.71	0.67/0.68
Random NFSRs	0.85/0.88	0.89/0.90	0.83/0.85

Given that secondary structures of NFSRs are predicted much more accurately than for FSRs, we determined whether the frequencies of specific discrepancies were similar to FSRs. Table 3 shows the frequencies for each type of these discrepancies for each secondary structure predictor. The distributions of these discrepancies differ significantly from FSRs for all three secondary structure predictors, with *p*‐values <2.4 × 10^−4^ in all three cases. Although H↔C discrepancies are most common for both NFSRs and FSRs, they are 15%–20% more common among NFSRs than FSRs (Tables 1 and 3). In contrast, H↔E and E↔C discrepancies are, respectively, 5%–9% and 10%–15% more common among FSRs. Thus, SS predictions with enriched H↔E and/or E↔C discrepancies could indicate fold switching more powerfully than SS predictions with more abundant H↔C discrepancies.

**Table 3:** Frequencies of Secondary Structure Discrepancy in Randomly Selected NFSRs

	JPred	PSIPRED	SPIDER2
H↔E	6%	3%	9%
H↔C	65%	69%	59%
E↔C	29%	28%	32%
*χ* ^2,*^	*p* < 1.0 × 10^−5^ (29.6)	*p* < 1.0 × 10^−5^ (53.6)	*p* < 2.4 × 10^−4^ (16.7)

^*^ *χ* ^2^ values were calculated by comparing frequencies of the three discrepancy types for FSRs and NFSRs for a given secondary structure predictor. *χ* ^2^ values are shown in parentheses.

### *Secondary structure prediction inaccuracies in FSRs*

It is perhaps unsurprising that secondary structure predictions of FSRs are significantly less accurate than NFSRs. By definition, FSRs have two distinct secondary structure configurations. JPred, PSIPRED, and SPIDER2 are designed to predict only one, best‐guess secondary structure configuration for a given amino acid sequence, however. Thus, these predictors could at best correctly predict half of the FSR configurations: it is impossible for them to predict both configurations with high accuracy. It is possible, however, for these algorithms to predict both FSR configurations inaccurately. To explore whether secondary structure prediction algorithms tend to predict one FSR conformation with high accuracy and the other with moderate‐to‐low accuracy or both conformations with moderate‐to‐low accuracies, we grouped FSR pairs into two categories: one where at least one of the two FSR conformations had a *Q*_3_ value ≥0.8 and one where both *Q*_3_ values were <0.8. Table 4 shows that accurate prediction of at least one conformer occurs for approximately half of FSR pairs for JPred and PSIPRED, and 36% of pairs for SPIDER2. Prediction accuracies of the remaining fold‐switch pairs were moderate‐to‐low (*Q*_3_ scores <0.8 for both conformers.)

**Table 4:** Fractions of Fold Switchers with One Accurate Prediction (*Q*_3_ ≥ 0.8) or Two Inaccurate Predictions (*Q*_3_ < 0.8)

	JPred (%)	PSIPRED (%)	SPIDER2 (%)
*Q*_3_ ≥ 0.8	46	52	36
*Q*_3_ < 0.8	54	48	64

### *Conformational overrepresentation contributes to incorrect secondary structure predictions of FSRs*

Upon observing lower‐than‐expected secondary structure prediction accuracies of FSRs, we sought to determine their source. We hypothesized that FSRs with many solved structures in the PDB were more likely to be predicted accurately, while FSRs with fewer representative structures were more likely to be predicted inaccurately. This hypothesis was based on the fact that these three secondary structure predictors are trained on proteins with solved structures. Thus, training sets would more likely contain protein conformations that were highly represented within the PDB. Alternatively, the prediction bias might result from differences in the algorithms' performance on the distinct secondary structures, for instance.

To test our hypothesis that FSR conformations with more solved structures have more accurate secondary structure predictions, we determined the number of structures available for each FSR conformation and tested if the more accurate of the two FSR predictions for each fold‐switched conformer tended to have at least as many, if not more, representative structures in the PDB than the conformer predicted less accurately. Our null hypothesis was that FSRs were equally likely to be predicted correctly, regardless of how many PDB structures represented them. By performing the binomial test, we disproved the null hypothesis [*p*‐values of 4.0 × 10^−6^ (JPred), 1.5 × 10^−4^ (PSIPRED), and 5.0 × 10^−3^ (SPIDER2)], demonstrating that secondary structure predictions are biased toward predicting FSR conformations with frequent PDB representation and biased against predicting FSR conformations with infrequent PDB representation.

## Discussion

Although most proteins with solved structures adhere to the classical notion that proteins adopt one secondary structure scaffold that performs one specific function, there are a number of exceptions.^5^, ^11^ Such exceptions include fold‐switching proteins, which remodel their secondary structures in response to cellular stimuli, fostering changes in function or enabling tight cellular control. Because fold‐switching proteins do not conform to the classical notion, their dual conformations and functionalities are unlikely to be recognized by current homology‐based secondary structure predictors, which are largely trained on the structures of classically folded proteins. Here, we seek to leverage this observation by using inaccurate secondary structure predictions to identify potential FSRs within proteins. Previous efforts have identified flexible regions in proteins by identifying inconsistent secondary structure predictions among different predictors, though on a limited dataset.^26^

Here we show that poor secondary structure prediction accuracies (*Q*_3_ < 0.6) can indicate that a protein (or region therein) switches folds. We first showed that secondary structure predictions that are inconsistent with experimentally determined protein structures are significantly more common in FSRs than in NFSRs, especially at low levels of accuracy. Given that secondary structure predictors cannot predict the two distinct secondary structure configurations of FSRs, this observation is not surprising, but it gives statistical power to our observation that secondary structure predictions of FSRs are often inconsistent with experiment.^5^ Thus, these differences can be used as a preliminary indicator of fold switching that relies on amino acid sequence and secondary structure annotations of one solved structure.

Although secondary structure prediction discrepancies are a good preliminary indicator of fold switching, they are not definitive, especially when *Q*_3_ accuracies exceed 0.6. Other factors—such as independent folding cooperativity—appear necessary for proteins to switch folds.^5^ Furthermore, secondary structure prediction algorithms depend heavily on the available amino acid sequences of proteins with solved structures. Our results suggest that this dependence can lead to prediction bias when one configuration of a fold‐switching protein is overrepresented relative to another.

As other intrinsic physical properties of fold‐switching properties are identified, robust physically based predictions of fold switchers could be developed to circumvent the limitations of homology‐based predictions. For example, the prediction accuracies and biases of all three homology‐based secondary structure predictors were similar (Tables 2 and 3), indicating common strengths and weaknesses among all approaches. In contrast, physically based predictions would not be similarly misled by biased representation of protein structures in the PDB. Nevertheless, we used homology‐based secondary structure predictions because they are the current state‐of‐the‐art. For example, when the sequence of a protein of interest falls below the threshold of significant similarity,^27^ these sequence‐based secondary structure predictions remain a viable option for model building. In fact, homology‐based secondary structure predictions indicated the functions of unannotated archaeal genes with surprising robustness^28^; indeed, secondary structure was the best predictor of all sequence properties tested.

Accurate predictions of fold switching could suggest biological mechanisms underlying observed experimental phenomena. For example, some proteins can change their cellular localizations by switching folds.^29^, ^30^ Others require fold switching to control their function(s).^7^, ^23^ Thus, predictions suggesting that a protein switches folds could lead to the identification of mechanisms underlying unexplained biological processes. Furthermore, fold‐switching proteins could constitute promising drug targets if their conformational equilibria could be disrupted by small molecules. Consistent with this notion, the veterinary medicine halofuginone arrests growth of the malaria parasite through stabilizing its prolyl tRNA synthetase in an inactive configuration.^31^, ^32^ Thus, predicting whether a protein switches folds could foster the discovery of new biological processes and drug discovery paradigms. We hope our results will lay groundwork for these advances.

## Methods

### *Secondary structure predictions of FSRs*

All amino acid sequences of 192 fold‐switching protein structures,^5^ corresponding to two different conformations of 96 fold‐switching proteins (96 proteins, two structures each; aka fold‐switch pairs), were downloaded from the PDB^33^ and saved as individual FASTA^34^ files. Separate secondary structure predictions were run on each file using JPred4, PSIPRED, and SPIDER2. JPred4 predictions were run remotely using a publicly downloadable scheduler available on the JPred4 website.^19^ PSIPRED and SPIDER2 calculations were run locally using the nr database.^35^ Secondary structure predictions from .jnetpred (JPred), .horiz files (PSIPRED), and .spd3 files (SPIDER2) were converted into FASTA format. Each residue was assigned one of three secondary structures: “H” for helix, “E” for extended β‐strand, and “C” for coil. Experimentally determined and predicted secondary structures that were neither helix nor extended were classified as coil (including β‐turns), except for chain breaks, which were annotated “‐.” The maximum allowable sequence length for JPred predictions is 800 residues. Sequences that exceeded this length were pruned before being submitted to JPred only; pruning occurred on the N‐terminus, C‐terminus, or both N‐ and C‐termini depending on whether the FSR was nearer to the C‐terminus, N‐terminus, or middle of the protein, respectively.

### *Secondary structure prediction accuracy calculations*

Secondary structure prediction accuracies were calculated using the *Q*_total_ (or *Q*_3_) metric,^22^ in which predicted and experimentally determined secondary structures are compared one‐by‐one, residue‐by‐residue. Predictions were scored as follows: (in)consistent pairwise predictions were given a score of (0)/1, summed, and normalized by the length of the sequences compared. Chain breaks were excluded from both scoring and normalization. Sequences composed of ≥10% chain breaks or more were excluded from calculations. *Q*_3_ values are typically expressed as decimals; occasionally we express the *Q*_3_ value as a percentage and refer to it as an accuracy.

### *Secondary structure prediction accuracy distributions*

Prediction accuracy distributions were calculated on FSRs, as defined.^5^ Window size equaled the length of the FSR, unless it fell below 40 residues, the minimum length required for secondary structure predictions. FSR lengths below this minimum were padded symmetrically or as symmetrically as possible if located near a terminus. FSRs of solved protein structures were identified using the pairwise2.align.localxs function from Biopython^36^ with gap‐forming score of −1 and gap‐elongation score of −0.5. Distributions in Figure 2 were plotted using Matplotlib.^37^

### *Randomly generated secondary structure predictions*

First, we used the procedure described in Secondary structure predictions of FSRs to predict the structures of 226 proteins with high likelihood of not switching folds.^5^ Segments were selected from 10 random regions of each protein. Segment lengths were randomly selected from the distribution of FSR lengths from the 192 proteins described previously. Secondary structure prediction accuracies were calculated using the *Q*_3_ metric,^22^ comparing predicted and experimentally determined secondary structures.

### *Kolmogorov–Smirnov (KS) statistics and PDB bias*

We found the KS test to give implausibly low *p*‐values for large distributions. To minimize this effect, we used the size of the smaller distribution twice, instead of using the sizes of the smaller and larger distributions once each.

To determine PDB bias, we BLASTed the sequences of our 192 proteins against the PDB (e‐value threshold: 1e‐04) and compared the structures of all hits with the structures of both fold‐switch‐pair conformations. Hits were grouped with the FSR‐pair conformation to which they had the highest secondary structure similarity, as indicated by the *Q*_3_ score. PDBs deposited before 7/27/17 were considered, and their secondary structures were determined using DSSP.^38^ All three secondary structure predictors were trained on structures deposited before this date.
